# Maml1 acts cooperatively with Gli proteins to regulate sonic hedgehog signaling pathway

**DOI:** 10.1038/cddis.2017.326

**Published:** 2017-07-20

**Authors:** Roberta Quaranta, Maria Pelullo, Sabrina Zema, Francesca Nardozza, Saula Checquolo, Dieter Matthias Lauer, Francesca Bufalieri, Rocco Palermo, Maria Pia Felli, Alessandra Vacca, Claudio Talora, Lucia Di Marcotullio, Isabella Screpanti, Diana Bellavia

**Affiliations:** 1 Department of Molecular Medicine, Sapienza University, Rome 00161, Italy; 2 Department of Medico-Surgical Sciences and Biotechnologies, Sapienza University, Latina 04100, Italy; 3 Center for Life Nano Science@Sapienza, Istituto Italiano di Tecnologia, Rome 00161, Italy; 4 Department of Experimental Medicine, Sapienza University, Rome 00161, Italy; 5 Institute Pasteur-Foundation Cenci Bolognetti, Sapienza University, Rome 00161, Italy

## Abstract

Sonic hedgehog (Shh) signaling is essential for proliferation of cerebellar granule cell progenitors (GCPs) and its misregulation is linked to various disorders, including cerebellar cancer medulloblastoma. The effects of Shh pathway are mediated by the Gli family of transcription factors, which controls the expression of a number of target genes, including *Gli1*. Here, we identify Mastermind-like 1 (Maml1) as a novel regulator of the Shh signaling since it interacts with Gli proteins, working as a potent transcriptional coactivator. Notably, Maml1 silencing results in a significant reduction of Gli target genes expression, with a negative impact on cell growth of NIH3T3 and *Patched1*^*−/−*^ mouse embryonic fibroblasts (MEFs), bearing a constitutively active Shh signaling. Remarkably, Shh pathway activity results severely compromised both in MEFs and GCPs deriving from *Maml1*^*−/−*^ mice with an impairment of GCPs proliferation and cerebellum development. Therefore *Maml1*^*−/−*^ phenotype mimics aspects of Shh pathway deficiency, suggesting an intrinsic requirement for Maml1 in cerebellum development. The present study shows a new role for Maml1 as a component of Shh signaling, which plays a crucial role in both development and tumorigenesis.

Hedgehog (Hh) signaling has been implicated in the regulation of key events during developmental processes.^1^ Hh pathway is controlled by extracellular ligands (Sonic, Indian and Desert hedgehog) through interaction with the receptor Patched (Ptch), thereby enhancing Smoothened (Smo) function, which activates Gli transcription factors.^2^ Transcriptional activation is largely derived from Gli1 and Gli2, whereas Gli3 mainly shows repressor activity in the absence of ligand. Gli1, the final and strongest transcriptional activator,^3^ is both the downstream effector and a target gene of the pathway, representing a feedback loop that serves as a readout of Hh activity.^1, 4, 5^ Signaling through Smo causes nuclear translocation of Gli1, able to induce the expression of pro-proliferative target genes, including *Cyclins D1* and *D2* (refs 6,7), which directly promote the entry into the cell cycle and DNA replication. Sonic hedgehog (Shh) pathway has a pivotal role in controlling embryonic patterning and is a master regulator of cerebellar granule cell progenitors (GCPs) development.^8^

Cerebellar development is a finely orchestrated process that produces an elaborate set of folia separated by fissures. The process of foliation begins during the prenatal period with the formation of four principal fissures, which divide the cerebellum into five cardinal lobes.^9^ Shh secreted by Purkinje cells (PCs) from E17.5 onward in the mouse is a key GCPs mitogen that promotes proliferation^10, 11^ and sustains normal cerebellum foliation.^12, 13^ Especially, it has been shown that Shh signaling spatially and temporarily correlates with fissures formation, regulating the number of folia through its influence on GCPs expansion.^12^ Proliferation of granule cells and the process of cerebellar development appear to be strongly related to one another.^12, 14, 15^ Indeed, a deregulated Shh signaling alters the development of GCPs making them hyperproliferative and susceptible to malignant transformation into medulloblastoma (MB), the most frequent childhood brain tumor.^8, 16^ The biological and pathogenic importance of Shh signaling emphasizes the need to tightly control its action.

In this study, we identify Mastermind-like 1 (Maml1) as a novel regulator of Shh signaling. In mammals, Maml1 (refs 17,18) belongs to a family of proteins, also including Maml2 and Maml3 (refs 19,20), which act as transcriptional coactivators for Notch signaling,^21^ an evolutionarily conserved pathway.^22, 23, 24^ Maml1 has been recently shown to act as a coactivator in other cell signaling pathways, including p53 (ref. 25), MEF2C^26^ and *β*-catenin,^27^ in a Notch-independent manner. These findings suggest broader roles for Maml1 protein in regulating important physiological processes.

Here, we present evidence that Maml1 enforces the Shh pathway, via a novel Notch-independent mechanism. At the molecular level, we found that Maml1 physically interacts with Gli1 and Gli2, promoting Shh-dependent transcriptional events. In addition, we show that Maml1 silencing disrupts Shh signaling with a significant reduction of Gli target genes expression. Noteworthy, in mouse embryo fibroblasts (MEFs) and GCPs deriving from *Maml1*^*−/−*^ mutant mice, the Shh pathway is strongly compromised, resulting in a decreased expression of Gli1 and Gli2, which impacts on GCPs proliferation and cerebellum development.

## Results

### Maml1/Gli1 protein–protein interaction reinforces the activation of Shh target genes

**Figure 1:**
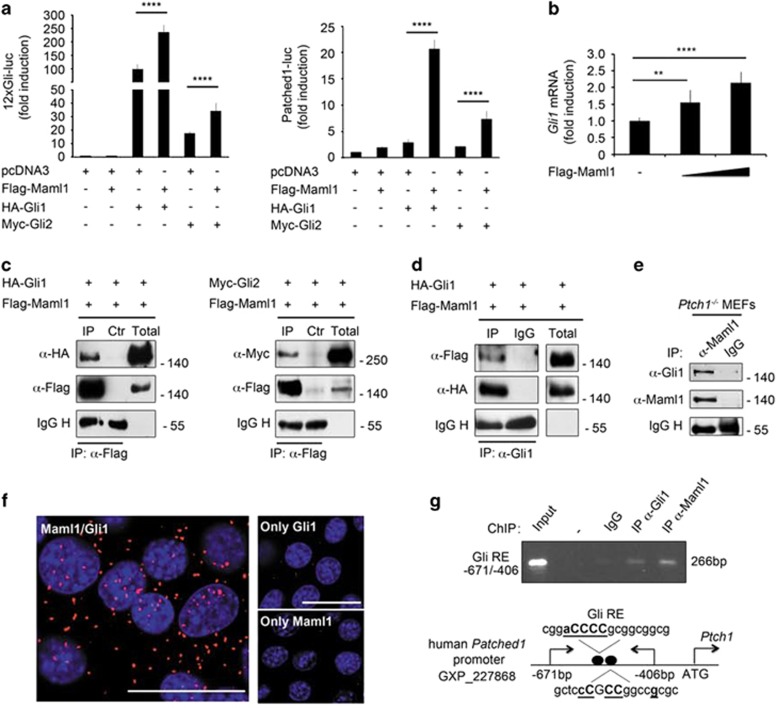
Maml1 interacts with Gli proteins and sustains Gli-mediated transcription. (**a**) Luciferase assay in HEK293T cells transfected with 12xGli-luc (left panel) or Patched1-luc (right panel) reporter and different combinations of plasmids encoding for Maml1, Gli1 and Gli2 as indicated. Luciferase activity is expressed as fold induction relative to pcDNA3 alone (empty control). (**b**) Endogenous *Gli1* mRNA levels evaluated by qRT-PCR in NIH3T3 cells transfected with increasing amounts of Maml1, compared with pcDNA3. (**c**) Coimmunoprecipitation (co-IP) experiments performed in HEK293T cells, transfected with expression vectors encoding for the indicated tagged proteins and immunoprecipitated with anti-Flag without (IP) or with (Ctr) blocking peptide. The interaction with Gli1 and Gli2 was revealed with anti-HA (left panel) or anti-Myc (right panel), respectively. (**d**) Reciprocal co-IP was performed by using anti-Gli1 antibody or control mouse antisera (IgG) and the interaction with Maml1 was detected with anti-Flag. Gli1 and Maml1 protein levels in total cell lysates are shown. (**e**) *Ptch1*^*−/−*^ MEFs were lysed and immunoprecipitated with Maml1 antibody or control rabbit antisera (IgG) and the blot was reprobed with Gli1. (**f**) Representative immunofluorescence images of endogenous Gli1/Maml1 interaction in NIH3T3 cells analyzed by *in situ* proximity ligation assay (PLA). Negative controls lacking one of the primary antibodies. No significant fluorescent signal was detected in NIH3T3 cells incubated with only one primary antibody (only Gli1; only Maml1). Single plane confocal images were captured using a 60 × oil objective. Protein complexes were visualized in red; nuclei were DAPI labeled (blue). Scale bar: 100 *μ*m. (**g**) ChIP assay in HEK293T cells. PCR was performed using primers that amplify Gli consensus binding sites on human *Ptch1* promoter (upper panel) as shown in schematic representation (lower panel). According to Genomatix, basepairs in bold and underlined are important, since they appear in a position where the matrix exhibits a high conservation profile (ci-value>60); basepairs in capital letters denote the core sequence used by MatInspector. Dark circles represent predict binding sites. Data reported as mean±S.D. **P≤0.01; ****P≤0.0001

Based on Maml1 functions as a transcriptional coactivator in several signaling pathways^28^ and being its expression significantly higher in the cerebellum than in other tissues (Supplementary Figure S1a), we sought to examine Maml1 role in the activation of the Shh pathway. To address this issue, HEK293T cells were cotransfected with 12xGli-luc (an artificial Gli reporter containing 12 copies of Gli-responsive elements) or Patched1-luc and vectors expressing Gli1 or Gli2 alone and in combination with Maml1. Figure 1a shows that Maml1 strongly cooperates with Gli proteins to potentiate both of Shh-responsive reporters. Moreover, these results confirm that Gli1 is a stronger transactivator than Gli2, also in the presence of Maml1 (ref. 3). We examined whether Maml1 sustains the endogenous Gli1 transcriptional activity, the readout of Shh activation pathway. Therefore, we monitored Gli1 expression in NIH3T3 cells transfected with Maml1. Figure 1b shows increasing expression levels of endogenous *Gli1* in a dose-dependent manner. The cooperation of Maml1 with Gli1 or Gli2 function suggests their physical association. Figure 1c shows Gli1 and Gli2 in Maml1 immunoprecipitates, indicating the formation of Maml1/Gli complexes, confirmed also by reciprocal coimmunoprecipitation assay using anti-Gli1 antibody (Figure 1d). To demonstrate endogenous Gli1/Maml1 protein interaction, we used Maml1 immunoprecipitates from *Ptch1*^*−/−*^ MEF cells, with a constitutively active Shh pathway (Figure 1e).^29^ Figure 1f also reveals the endogenous Gli1/Maml1 complex in NIH3T3 cells by *in situ* proximity ligation assay (PLA), detecting single interaction pairs of native proteins by using antibodies directed against Maml1 and Gli1. Only interacting proteins pairing displays a red signal by confocal microscopy. Indeed, a high degree of Maml1/Gli1 interaction was observed both in cytoplasmic and nuclear compartment. To determine the occupancy of the Maml1/Gli complex on Shh target genes, we performed a chromatin immunoprecipitation (ChIP) assay. Figure 1g (upper panel) shows that both Maml1 and Gli1 are recruited at the same Gli binding sites in the human *Patched1* promoter, as shown in the schematic representation of Figure 1g (lower panel). These data indicate that Maml1 physically interacts with Gli proteins and cooperatively they activate specific Shh-responsive target genes.

### Maml1 C-terminal region is required to allow its cooperation with Gli1

**Figure 2:**
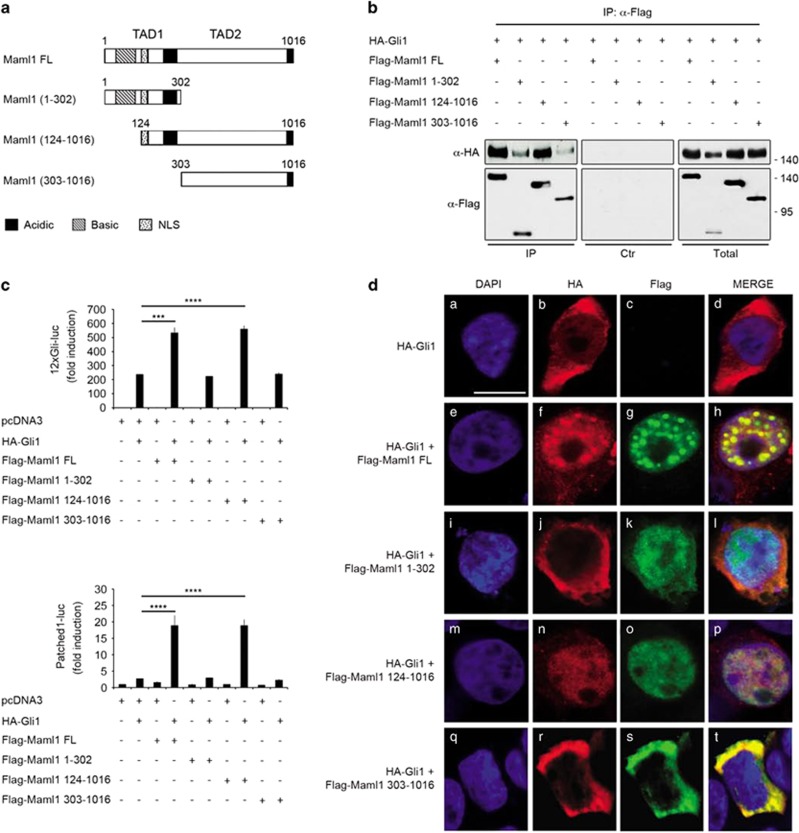
Maml1 COOH-terminus plays a functional role on Gli1 activity. (**a**) Schematic representation of full-length (FL) and truncated Maml1 constructs. Transcription activation domain (TAD): TAD1 (75–301 aa); TAD2 (303–1016 aa). (**b**) Whole-cell extract (WCE) from HEK293T cells cotransfected with indicated expressing vectors were immunoprecipitated with anti-Flag (IP) or anti-Flag with blocking peptide (Ctr). Immunoprecipitates and aliquots of cell lysates (Total) were immunoblotted with the indicated antibodies. (**c**) Luciferase assays of cotransfected HEK293T with 12xGli-luc (upper panel) or Patched1-luc (lower panel) and different combinations of expression vectors, as indicated. Luciferase activity is expressed as fold induction relative to control (pcDNA3). Data represent mean±S.D. ***P≤0.001; ****P≤0.0001. (**d**) Representative single plane confocal immunofluorescence images of HEK293T cells cotransfected with indicated expression vectors. Flag- (green) or HA- (red) tags were visualized by confocal microscopy. Confocal images were captured using a 60 × oil objective. Nuclei were DAPI labeled (blue). Scale bar: 10 *μ*m

To examine which region of Maml1 interacts with Gli, we used truncated mutant Maml1 proteins (schematic representation in Figure 2a) to perform coimmunoprecipitation assays. We focused on Gli1, the most powerful effector of the Shh pathway, which is able to enhance its own expression, autoreinforcing the signaling strength. We show in Figure 2b that Gli1 is able to bind both the N-terminal region (amino acids 1–302) and the C-terminal region (amino acids 303–1016) of Maml1 protein, independently. This observation suggests that Gli1 binds Maml1 at least in two distinct domains. To determine which region of Maml1 is important for the transcriptional activity of Gli1, we cotransfected HEK293T cells with the combination of Gli1, Maml1 mutant forms and 12xGli- or Patched1-luc reporter constructs. Figure 2c shows that Maml1 full-length (FL) and Maml1 124–1016 strongly enhance luciferase reporter gene activity in the presence of Gli1 vector either on 12xGli-promoter (upper panel) or Patched1 promoter (lower panel). Conversely, the Maml1 COOH-terminal deleted mutant (1–302) and the Maml1 303–1016 without the nuclear localization signal (NLS) have no detectable effect on Gli1 transcriptional activity. Interestingly, the Maml1 FL protein has been previously shown to drive Maml1-interacting proteins, such as Notch,^17, 30^ p300 (ref. 31), MEF2C^26^ and GSK3*β*^32^ to the nucleus, in particular into nuclear bodies.^32^ To this regard, Maml1 mutant proteins appeared to exert a distinct influence on Gli1 subcellular localization. In fact, Figure 2d shows that Maml1 FL is able to address Gli1 into the nucleus, particularly into nuclear bodies (panel h *versus* d). Instead, in cells transfected with Maml1 124–1016, Gli1 completely diffuse into the nucleus (panel p). Contrarily, Maml1 1–302 is not able to address Gli1 into the nucleus (panel l) and Maml1 303–1016, deleted of the NLS domain, sequesters Gli1 into the cytoplasm, where the two proteins preferentially interact (panel t). Subcellular distribution of transiently transfected Flag-tagged Maml1 FL and mutant forms is shown in Supplementary Figure S2. Overall the data suggest that Maml1 is required to sustain the nuclear localization of Gli1, as further supported by the nuclear and cytoplasmic fractionation assay (Supplementary Figure S3), and that Maml1 COOH-terminal region is required to reinforce the transcriptional activity of Gli1 *in vitro*.

### Maml1 is required to fully activate Gli-mediated target gene transcription

**Figure 3:**
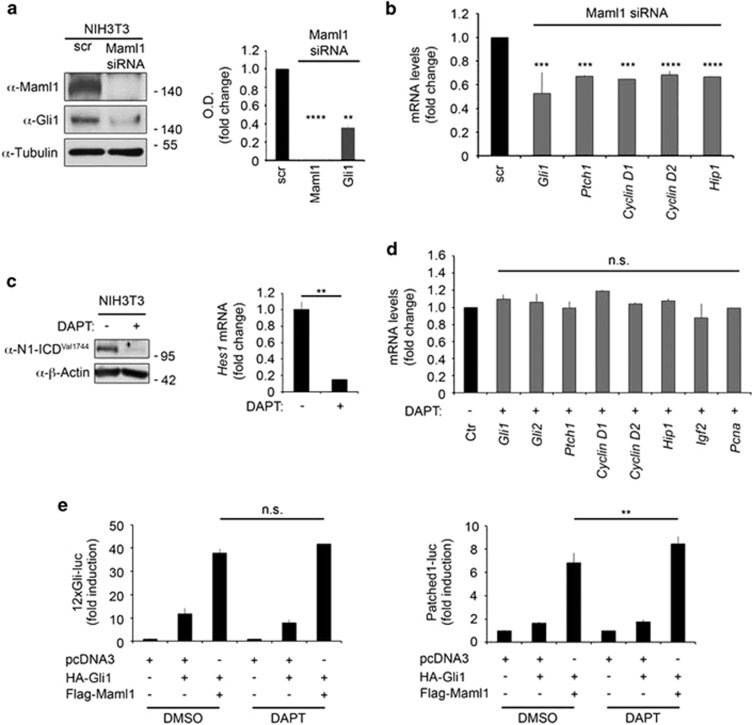
Maml1 cooperates with Gli1 in a Notch-independent manner. (**a**) Proteins expression was detected by immunoblot analysis as indicated in WCE derived from NIH3T3 cells transfected with Maml1 siRNA or control siRNA (scr). Anti-tubulin was used as a loading control (left panel). Optical densitometry (OD) analysis of Maml1 and Gli1 protein expression (right panel). (**b**) qRT-PCR analysis of Shh target genes in NIH3T3 48 h after silencing of Maml1 compared with control (scr). (**c**) WCE separated by SDS-page from NIH3T3 upon 72 h treatment with DAPT or control vehicle (DMSO). To reveal the activated form of Notch1, an anti-Notch1 Val 1744 antibody was used (left panel). Expression level of *Hes1* mRNA was evaluated by qRT-PCR (right panel). (**d**) Expression levels of Shh target genes evaluated by qRT-PCR in DAPT or DMSO (Ctr) treated NIH3T3 cells. (**e**) Luciferase assays in HEK293T cells cotransfected with 12xGli-luc (left panel) or Patched1-luc (right panel) and different combinations of expression vectors as indicated. After DAPT or control vehicle treatment, cell lysates were harvested for luciferase assay. Data represent mean±S.D. NS, not significant; **P≤0.01; ***P≤0.001; ****P≤0.0001

Overall, our data suggest that Maml1 influences Gli1 subcellular localization and acts as a transcriptional coactivator strengthening the expression of Gli target genes. To further support the model, we analyzed whether Maml1 loss of function influences the transcriptional activity of endogenous Gli1. Importantly, the small interference RNA (siRNA)-mediated depletion of Maml1 induces a significant impairment of Gli1 protein expression (Figure 3a) and Shh target genes, as *Gli1* itself, *Ptch1*, *Cyclin D1, Cyclin D2* and *Hip1* (Figure 3b).

Notably, Maml1 is a well-known transcriptional coactivator of the Notch pathway and to exclude the possibility that Notch signaling impairment might be responsible of the observed effects, we investigated the expression of Hh target genes in the presence of the *γ*-secretase inhibitor (DAPT) that blocks the Notch pathway activation. We treated NIH3T3 cells with DAPT that affects Notch1 activation, as revealed by using the antibody against the valine 1744 (N1ICD^Val1744^) (Figure 3c, left panel), and down-modulates its target gene, *Hes1* (Figure 3c, right panel). In contrast, quantitative real-time PCR (qRT-PCR) assays reveal that *Gli1* itself and Shh target gene expression levels are not significantly modified by Notch inhibition (Figure 3d), indicating that the role of Maml1 on Hh/Gli1 signaling is independent of the Notch activity. Accordingly, Maml1 potently enhances Gli1 activity upon cotransfection with 12xGli-luc (Figure 3e, left panel) or Patched1-luc (Figure 3e, right panel), independently from the presence of DAPT. Together these findings directly and functionally connect Maml1 to the Shh pathway, suggesting a Maml1-dependent reinforcement mechanism of Gli1 transcriptional activity in a Notch-independent manner.

### *Maml1* deletion results in an impaired Shh signaling cascade

**Figure 4:**
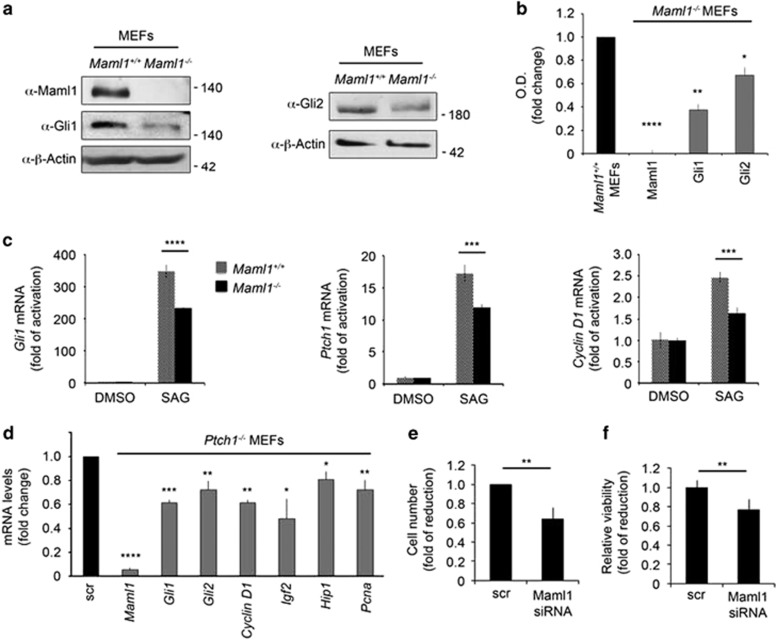
Maml1 loss of function determines an impaired Shh signaling pathway. (**a**) Immunoblot analysis of WCE from *Maml1*-deficient (*Maml1*^*−/−*^) and control (*Maml1*^*+/+*^) MEFs, using the indicated antibody. (**b**) Optical densitometry (OD) analysis of Maml1, Gli1 and Gli2 protein expression evaluated by immunoblotting, related to panel (**a**). (**c**) Representative qRT-PCR analysis of Hh target genes in *Maml1*^*−/−*^ MEFs compared with control upon SAG treatment for 48 h. The data are presented as fold of activation respect to DMSO. (**d**) qRT-PCR analysis of *Maml1* mRNAs and Shh target genes in *Ptch1*^*−/−*^ MEF cells transfected with Maml1 siRNA or control siRNA (scr) for 48 h. (**e**) Trypan blue cell counting to determine the rate of proliferation and the number of viable cells in *Ptch1*^*−/−*^ MEFs after transfection with Maml1 siRNA for 48 h, compared with scramble (scr). (**f**) MTT cell proliferation assay in *Ptch1*^*−/−*^ MEF cells Maml1-silenced compared with control. The data are presented as fold of reduction respect to scramble. Data represent mean±S.D. **P≤0.01; ***P≤0.001; ****P≤0.0001

To further validate that Maml1 functions as a coactivator of Gli transcription factors, we used *Maml1*^*−/−*^ MEFs^33^ model. Interestingly, the absence of *Maml1* gene determines a significant reduction of endogenous Gli1 (Figures 4a, left panel and b) and Gli2 (Figures 4a, right panel and b) expression levels in *Maml1*^*−/−*^ MEFs, compared with control. Moreover, the absence of Maml1 determines an impaired activation of Shh signaling in *Maml1*^*−/−*^ MEFs in response to treatment with the Smo agonist, SAG.^34^ Figure 4c shows an important decrease of Shh target genes expression, such as *Gli1* itself, *Ptch1* and *Cyclin D1*, in *Maml1*^*−/−*^ *versus Maml1*^+/+^ SAG-treated MEFs. Supplementary Figure S4 also shows reduced Gli1 expression levels upon SAG treatment in *Maml1*^*−/−*^ respect to control, in a time-dependent manner. To investigate the requirement of Maml1 for Gli function, we used the *Ptch1*^*−*/*−*^ MEFs, in which *Ptch1* deletion causes constitutive Gli activation. Notably, the Maml1 silencing impairs Shh signaling, by promoting the inhibition of *Gli1* and *Gli2* and specific Shh target genes (Figure 4d), associated with a decreased proliferation rate (Figure 4e), also revealed by MTT assay (Figure 4f). These data further demonstrate that Maml1 is functionally required to sustain full activation of Shh signaling, by acting as a crucial coeffector also when the pathway is constitutively activated.

### Reduced GCPs proliferation correlates with a decreased Gli activity in *Maml1*^*−/−*^ mice

**Figure 5:**
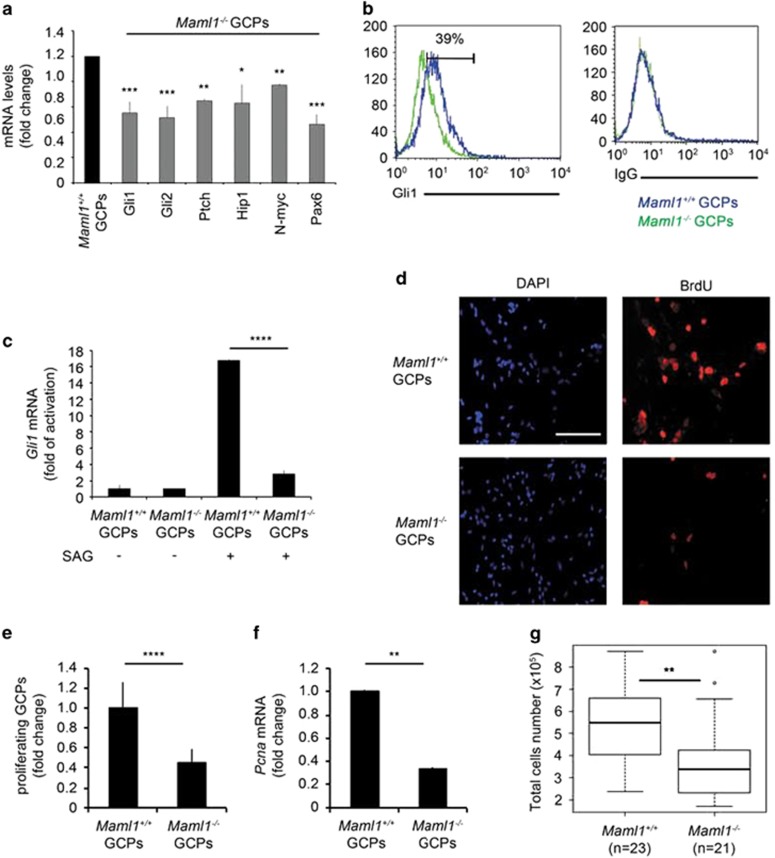
Reduced GCPs proliferation correlates with impaired expression of Shh target genes in *Maml1*^*−/−*^ mice. (**a**) qRT-PCR analysis shows mRNA expression level of Shh target genes in GCP cultures from *Maml1*^*−/−*^ and control mice. (**b**) GCPs from E19.5 *Maml1*^*+/+*^ (blue) and *Maml1*^*−/−*^ (green) mice were analyzed by flow cytometry analysis with a specific anti-Gli1 antibody or IgG, used as a isotype control. (**c**) GCPs from E19.5 *Maml1*^*−/−*^ and control littermates cerebella were treated with SAG or vehicle alone (DMSO) for 24 h. Endogenous *Gli1* mRNA expression level was determined by qRT-PCR analysis. The results were analyzed as fold of activation, compared with control (DMSO). (**d**) BrdU incorporation assay in SAG-treated *Maml1*^*+/+*^ and *Maml1*^*−/−*^GCPs at E19.5 after a 24 h BrdU pulse. Proliferating cells are visualized in red; nucleus was labeled in blue. Scale bar: 200 *μ*m. (**e**) Mitotic index was calculated by number of BrdU-positive GCPs/total GCPs. The results were analyzed as fold of reduction respect to control cells. (**f**) qRT-PCR analysis of *Pcna*, an Shh target involved in cellular proliferation, in *ex vivo* GCP cultures from *Maml1*^*−/−*^ and control mice. (**g**) Trypan blue cell counting was performed to estimate the growth rate of viable cells obtained from GCP cultures from *Maml1*^*+/+*^ (*n*=23 mice) and *Maml1*^*−/−*^ (*n*=21 mice) at E19.5. The box plot shows the distribution of cellular counts. Data represent mean±S.D. *P≤0.05; **PP≤0.01; ***PP≤0.001; ****P≤0.0001

Shh signaling is a master regulator of the development of cerebellar GCPs.^8^ To investigate the relevant role of Maml1 in sustaining Shh signaling in GCP cells, we measured endogenous Shh/Gli1 target genes in primary GCPs, derived from *Maml1*^*−/−*^ and control mice, by qRT-PCR analysis. *Maml1*^*−/−*^ mice were studied up to E19.5, since they die at perinatal period. Figure 5a shows that the absence of Maml1 determines a remarkable reduction of Gli1 target genes in *Maml1*^*−/−*^ GCPs when compared with *Maml1*^*+/+*^ littermates, associated with an important decrease of Gli1 protein expression (∼39%) (Figure 5b). To further investigate whether loss of Maml1 activity might result in a defective Shh signaling, we measured the expression of the direct Shh target gene *Gli1* by qRT-PCR, upon SAG stimulation. Figure 5c shows that the Gli1 activity is significantly reduced in GCPs of *Maml1*^*−/−*^, when compared with wild-type (wt) mice.

Shh signaling supports the proliferation of GCPs during cerebellar development.^12, 14^ Thus, we examined whether the absence of Maml1 can antagonize the mitogenic effect of Shh on GCPs proliferation. For this purpose, we cultured GCPs deriving from *Maml1*^*−/−*^ and wt littermates, after SAG treatment and pulsed with bromodeoxyuridine (BrdU) to label proliferating cells. Intriguingly, only a small percentage of *Maml1*^*−/−*^ GCPs incorporates BrdU (Figure 5d), with nearly 3-fold decrease in BrdU-positive cell number (Figure 5e). Moreover, Figure 5f shows a reduced endogenous *Pcna* (proliferating cell nuclear antigen) expression in *Maml1*^*−/−*^ GCPs, which correlates to a decrease in number of total cerebella-derived GCPs in E19.5 *Maml1*^*−/−*^ mice (Figure 5g).

To discriminate the role sustained by Shh and Notch signaling in GCPs, we performed specific pharmacological treatments in order to compare the outcomes of these signaling pathways on cerebellar progenitors proliferation. Firstly, we evaluated the expression of Notch pathway genes in GCPs. Supplementary Figure S5a shows that Notch receptors and specific target genes are similarly expressed in *Maml1*^*−/−*^ and *Maml1*^*+/+*^ GPCs, except for Hes1, which appears decreased in *Maml1*^*−/−*^. Noteworthy, Hes1 has been described to be also an Shh signaling target.^35^ Then, we performed BrdU assay in SAG-activated *Maml1*^*+/+*^ GCPs, treated with DAPT or KAAD/cyclopamine.^36^ Interestingly, Supplementary Figures S5b and c show that KAAD/cyclopamine treatment impairs GCPs proliferation with a significant effect, when compared with DAPT, and this is associated with an important downregulation of Shh/Gli1 target genes (panel d).

Furthermore, Supplementary Figure S6a shows that the proliferation of DAPT-treated *Ptch1*^*−*/*−*^ MEFs, revealed by MTT analysis, is more preserved, when compared with KAAD/cyclopamine treatment, suggesting that Notch pathway plays a marginal role in controlling the proliferation on Hh-activated cells. In addition, the inhibition of Notch pathway by DAPT does not influence the expression of Shh target genes (Supplementary Figures S6b and c), as instead does the KAAD treatment (Supplementary Figures S6b and d). Notably, Supplementary Figure S6b confirms *Hes1* as a common target gene of Notch and Shh pathways.^35^

### Cerebellar defects in *Maml1*^*−/−*^ mice

**Figure 6:**
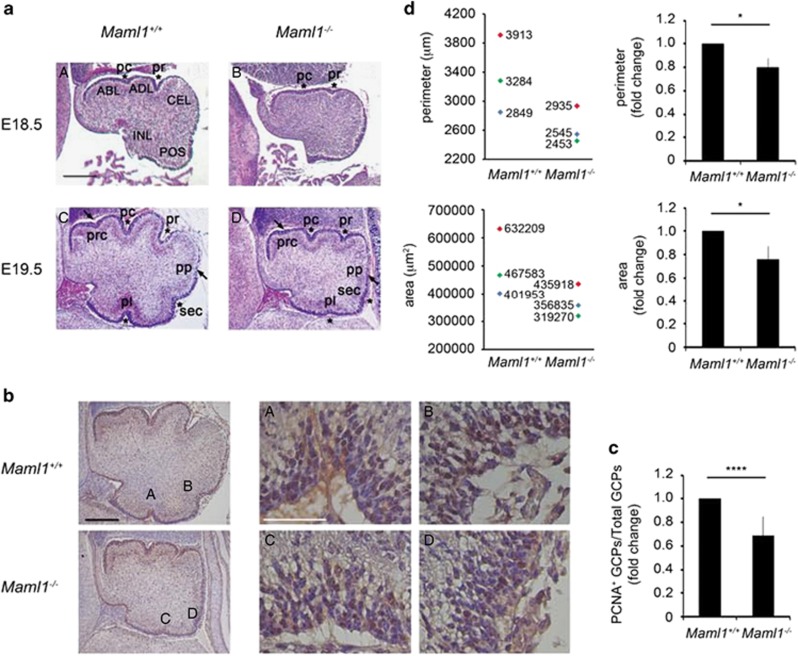
*Maml1*^*−/−*^ mutant mice present foliation defects in developing cerebellum. (**a**) Midsagittal sections of E18.5 and E19.5 *Maml1*^*−/−*^ and control cerebella stained with H&E. The four principal fissures (denoted by asterisks) as well as two additional fissures (indicated by the arrows) are shown in the figure. Abbreviations: prc, precentral; pc, preculminate; pr, primary; pp, prepyramidal; sec, secondary; pl, posterolateral fissures; ABL, anterobasal; ADL, anterodorsal; CEL, central; POS, posterior; INL, inferior lobes. Images of each panel were taken at the same magnification. Scale bar: 250 *μ*m. (**b**) Immunohistochemical staining for PCNA in midsagittal cerebellar sections from *Maml1*^*+/+*^ and *Maml1*^*−/−*^ mice at E19.5 (left panels). Scale bar: 250 *μ*m. High magnification images of PCNA staining in the EGL, corresponding to posterolateral (A, C) and secondary (B, D) fissures (right panels). Scale bar: 50 *μ*m (**c**) Graph shows the number of PCNA-positive cells, analyzed as fold of reduction in comparison with *Maml1*^*+/+*^ control mice. Five sections/mouse *n*=3 mice per group were analyzed. ****P≤0.0001 (**d**) Quantitative analyses of cerebellar morphology. Values of perimeter (upper) and area (lower) of midsagittal cerebella sections from three mice of each genotype are represented in the graphs (left panels). Littermates are indicated with the symbol of the same color. The histograms (right panels) show *Maml1*^*−/−*^ cerebellum perimeter (upper) and area (lower), represented as fold of reduction compared with control. *P≤0.05

Shh signaling plays a critical role and regulates the complexity of cerebellar foliation.^10, 14^ To establish whether the decreased proliferation observed in GCPs derived from *Maml1*^*−/−*^ mice may negatively impact on the foliation pattern, histological sections of cerebella at E18.5 and E19.5 were stained with hematoxylin and eosin (H&E). Figure 6a shows a defective foliation pattern in *Maml1*^*−/−*^ cerebella at E18.5 with slight indentations corresponding to preculminate (pc) and primary (pr) cardinal fissures; furthermore, the cardinal lobes are not identifiable, with respect to control (panel B *versus* A). Sagittal sections of cerebella at E19.5 in Figure 6a show that in wild-type mice the four principal fissures are formed, as well as two additional fissures. In contrast, E19.5 *Maml1*^*−/−*^ mice have only two primary fissures (pc and pr), although they are very shallow (panel D *versus* C). Of note, the posterolateral and secondary fissures are not visible, corresponding to regions with a highest Shh signaling.^12, 14^ Then, we performed immunostaining assay with PCNA, confirming that the cells from *Maml1*^*−/−*^ cerebellum are less mitotically active than in control (Figures 6b and c), in particular in correspondence of secondary and posterolateral fissures (C *versus* A and D *versus* B). The shallow principal fissures at E18.5 and delayed lobularization of *Maml1*^*−/−*^ cerebella are reminiscent of foliation defects observed in mice with altered Shh signaling.^13, 14^ In addition, morphometric analysis reveals that the perimeter and total area of *Maml1*^*−/−*^ cerebella are reduced, suggesting that Maml1 depletion negatively affects cerebellar size (Figure 6d). Overall, these results support our hypothesis that Maml1 protein is critical to mediate Shh signaling with an impact on cerebellum size and foliation during development *in vivo*.

## Discussion

Our findings unveil an additional mechanism of regulation of Shh pathway by identifying a novel role for Maml1 as a coactivator of Gli transcription factors. Maml proteins exhibit distinct expression patterns during embryonic development, supporting the hypothesis that they play specific roles in different tissues.^20, 37^ We analyzed Maml1 expression in Baseline Atlas^38^ and observed that the cerebellum is the district with the highest expression levels of Maml1, compared with other human tissues (Supplementary Figure S1a), suggesting an important role in this organ. Notably, using both *in vitro* and *in vivo* approaches, we found that Maml1 empowers Shh signaling, by modulating Gli1 and Gli2 activity. Noteworthy, coexpression of Maml1 and Gli1 or Gli2 considerably activates Gli target promoters, where Gli1 exhibits a stronger transcriptional activity than Gli2, also in the presence of Maml1. Moreover, increasing amounts of Maml1 reveal a significant dose-dependent upregulation of Gli1 transcripts, indicating that Maml1 is able to reinforce endogenous Shh pathway. In most of our experiments, we focused on Gli1, since it is the strongest transcriptional activator and the final effector of Shh signaling, being endogenously activated by Gli2 (refs 5, 39). Our results clearly show a robust synergistic association between Maml1 and Gli1 able to drive downstream transcriptional events of the Shh signaling cascade. Significantly, Maml1 loss of function in NIH3T3 or in constitutively activated Shh pathway-bearing *Ptch1*^*−/−*^ MEFs determines a significant downmodulation of Gli target genes. This indicates that the Shh signaling activity is significantly downregulated in the absence of Maml1, with a negative impact on cell proliferation. Outstandingly, the absence of Maml1 significantly impairs the Shh signaling, as revealed not only by reduced expression levels of Gli1 and Gli2 in MEFs and GCPs from *Maml1*^*−/−*^ mice but also by a disrupted Gli activity when the pathway is activated, upon treatment with the specific Smo agonist, SAG.

Shh/Gli signaling represents the master regulator of the proliferation of GCPs.^11, 40^ Increasing data support the notion that Shh and its transcriptional mediators, Gli1 and Gli2, regulate not only GCPs proliferation *in vivo*, but also influence the foliation, the final size and the shape of the cerebellum lobes.^12, 13^ Shh expression from E17.5 to early postnatal stages is spatially restricted to the regions where fissures form first.^14^ Indeed, by removing Shh receptor, Smoothened or Gli1 and/or Gli2, the extent of foliation is significantly reduced, correlating with a decrease in GCPs proliferation. Mice lacking Gli1 appear phenotypically normal;^41^ however, the importance of this protein in cerebellum development is demonstrated by the observation that loss of Gli1 enhances the foliation defects of the Gli2-deficient cerebellum.^42^ Our data demonstrate that *Maml1*^*−/−*^ mice exhibit a disrupted Shh signaling with a significant downregulation of Gli1 and Gli2 and a reduced responsiveness to SAG. Our findings show that the impaired Shh signaling in *Maml1*^*−/−*^ GCPs results in a dramatic reduction of GCPs proliferation. Onward the decreased number of GCPs, we show an impaired foliation process in *Maml1*^*−/−*^ cerebella at E18.5 and E19.5, with almost complete lack of secondary and posterolateral fissures, corresponding to regions with the highest Shh signaling, characterized by fissures appearing very shallow. Therefore, *Maml1*^*−/−*^ phenotype is reminiscent of cerebella from mice with a reduced Shh signaling.^12, 14^ As GCPs expansion is a driving force of foliation,^15^ the reduced number of GCPs in *Maml1*^*−/−*^ cerebellum explains, at least in part, the foliation defects observed in these mice. Hence, Maml1 depletion severely perturbs Shh signal transduction and mimics aspects of Shh pathway deficiency with impaired GCPs proliferation, cerebellar foliation and size, suggesting an intrinsic requirement for Maml1 in cerebellum development. Our observations would support the notion that Maml1 is necessary to potentiate the Hedgehog pathway when a strong Hh signaling is required during development, as previously suggested in *Drosophila* follicle stem cells.^43^

Of note, the inhibition of Notch signaling pathway, using the *γ*-secretase inhibitor DAPT, does not affect the action of Maml1 on Gli1, suggesting that the role of Maml1 is Notch activation independent. Noteworthy, N-terminus of Maml1 is important for interaction with Notch and proteins of other signaling pathways, as MEF2C and p53 or with the coactivator p300. Conversely, we show that the N-terminal region of Maml1 is dispensable to sustain Gli1-mediated transcription, further supporting our hypothesis that the action of Maml1 on Gli1 is Notch-independent.

Notably, pharmacological inhibition of Shh and Notch signaling underlines different biological outcomes on GCPs and *Ptch1*^*−/−*^ MEFs, where Shh pathway shows a predominant effect in sustaining the proliferation compared with Notch signaling. Moreover, the observation that DAPT treatment is able to promote the Gli1 and Gli2 expression allow us to speculate that the Notch inhibition may indirectly regulates the Hh pathway by a dynamic competition for the same coactivator, Maml1. Overall these observations suggest that Maml1 represents a new link between Notch and Hh signaling pathways.

In conclusion, we provide a new integrated level of regulation in Shh/Gli pathway by identifying Maml1 as a novel coactivator that empowers Shh signaling with important implications on GCPs proliferation and an impact on cerebellum development.

Noteworthy, aberrant Shh pathway activation is associated with various disorders, including medulloblastoma, the most common brain tumor in childhood affecting cerebellum.^16, 44, 45, 46^ The *in silico* analysis reported in Differential Atlas database shows that *Maml1* expression increases in Shh-dependent medulloblastoma, when compared with healthy cerebellar tissue or other medulloblastoma subtypes (Supplementary Figure S1b). Thus, Maml1 could represent an attractive druggable target for future therapeutic approaches directed against Shh-driven tumors.

## Materials and methods

### Mice

The generation and typing of *Maml1*^*−/−*^ mouse have been described elsewhere.^33^ Mice were maintained on a C57BL/6 background; they were bred and held under specific pathogen-free conditions in animal facility. The studies involving animals have been conducted following the Italian national guidelines for use and care of experimental animals, established in D.Lgs. n.26/2014, and in accordance with European Directive 2010/63/UE.

### Cell culture, proliferation assay and treatments

NIH3T3, HEK293T and wild type and *Ptch1*^*−/−*^MEFs were maintained as described elsewhere.^47^ Primary wild type and *Maml1*^*−/−*^ MEFs were isolated from E13.5 littermates embryos, following the protocol from Xu.^48^ Primary granule cell precursor (GCPs) cells were cultured from E19.5 cerebella, according to established protocols^40, 49^ and after 3 h the medium was replaced for the starvation in serum-free medium and the cells were treated with 200 nM SAG or vehicle alone (DMSO). Cell proliferation was evaluated by BrdU-Labeling and Detection Kit (cat.#11296736001; Roche, Welwyn Garden City, UK), as previously described.^50^ Nuclei were counterstained with Hoechst reagent and images were acquired with an Axio Vert.A1 microscope and analyzed with Axio Vision LE64 Software (Carl Zeiss Microscopy GmbH, Jena, Germany). At least 500 nuclei were counted in triplicate and the number of BrdU-positive nuclei respect to total cells number was evaluated to calculate the proliferation rate. To analyze the cell growth rate in *Ptch1*^*−/−*^ MEFs, 2500 cells per well were plated onto a 96-well plate. The WST1 solution (cat.#5015944001; Roche) was added to each well according to the manufacturer’s instructions. Spectrophotometric absorbance at 450 nm wavelength was determined by the plate reader GloMax-Multi Detection System (Promega, Madison, WI, USA). Cells were treated with different compounds: 10 *μ*M *γ*-secretase inhibitor IX (DAPT, cat.#565770; Calbiochem, Merck Millipore, Darmstadt, Germany), 200 nM Smoothened agonist (SAG, cat.#ALX-270-426-M001; Enzo Life Sciences, Farmingdale, NY, USA) and 1 mM KAAD/cyclopamine (Shh pathway inhibitor) (cat.#239804; Calbiochem), for the times indicated in the figures. All compounds were dissolved in sterile DMSO, and DMSO was used in control treatments. Before SAG treatments, cell cultures were subjected to serum starvation.

### Cell transfection, luciferase assay and plasmids

Transient transfection of HEK293T and NIH3T3 cell lines were performed using Lipofectamine 2000 (Life Technologies, Carlsbad, CA, USA) or TransFectin Lipid Reagent (Bio-Rad, Hercules, CA, USA), according to the manufacturer’s instructions. Luciferase Assays were performed using the indicated reporter plasmids with different combinations of expression vectors, as shown in figures. pRL-TK Renilla was used as normalization control and pcDNA3 as control empty vector. Luciferase activity was assayed with a Dual Luciferase Assay system (Promega) as described previously.^51^ All luciferase activity data are presented as means±S.D. of value from at least three independent experiments, each performed in triplicate. The following plasmids were described elsewhere: human *Patched1* promoter (Patched1-luc) and promoter 12GLI-RETKO-luc (12xGli-luc);^52^ pCS2-HA3-Gli1 (ref. 53), pCS2-MT Gli2FL-Myc,^54^ pFLAG-CMV-2 Maml1 full-length (1–1016) (ref. 51), pFLAG-CMV-2 Maml1 (1–302) and pFLAG-CMV-2 Maml1 (124–1016) (ref. 17). cDNA corresponding to Maml1 303–1016 was amplified by PCR from pFLAG-CMV-2 Maml1 full-length (1–1016) and cloned as SalI/NotI fragment in pFLAG-CMV-2 (#E7033 Sigma-Aldrich).

### siRNA silencing

siRNA was performed using 100 nM SMART pool siRNA duplexes (cat.#L-059179-01-0005 for Maml1) or 100 nM scrambled control (cat.#D-001810-10-20) purchased by Dharmacon Inc. (Lafayette, CO, USA), using Lipofectamine RNAiMAX (Life Technologies), according to the manufacturer’s instructions.

### Reverse transcription PCR /qRT-PCR

Total RNA extraction and reverse transcription PCR (RT-PCR) were previously described.^55^ Extraction and reverse transcription of mRNA from GCPs was achieved through the use of Cells-to-CT 1-Step TaqMan Kit (Life Technologies, Carlsbad, CA, USA), according to the manufacturer’s instructions. Analysis of gene expression were realized by qRT-PCR using TaqMan designed assays on demand (Invitrogen, Life Technologies) for the specific target genes, reported in Supplementary Table S1, on the StepOnePlus Real-Time PCR System (Applied Biosystems, Life Technologies), following the manufacturer’s protocol for the comparative C_T_ method. mRNA quantification was expressed, in arbitrary units, as a ratio of sample quantity to the mean value of control sample. Normalization was carried out using hypoxanthine guanine phosphoribosyl transferase (*Hprt*) as internal control gene.

### Protein extract, immunoprecipitation and immunoblot analysis

Preparation of whole-cell lysates, fractionation of cytoplasmic and nuclear proteins and immunoprecipitation assays were performed as described elsewhere.^56^ Briefly, for coimmunoprecipitation in transfected HEK293T cells, cell lysate were incubated with agarose conjugate Flag M2 beads (cat.#A2220; Sigma-Aldrich, St. Louis, MO, USA) for 2 h at 4 °C. In control sample the antibody was saturated with anti-Flag peptide (cat.#F4799; Sigma-Aldrich). For reciprocal immunoprecipitation assay, after a pre-clearing step with Protein G-Agarose (cat.#sc-2002; Santa Cruz Biotechnology, Dallas, TX, USA), cell lysate was incubated with anti-Gli1 (C-1) (cat.#sc-515751; Santa Cruz Biotechnology) or normal mouse IgG (cat.#sc-2025; Santa Cruz Biotechnology) as control for overnight at 4 °C. The complexes were precipitated with Protein G-Agarose, then the beads were washed extensively with wash buffer and the interaction was evaluated by western blot analysis. Similarly, for *Ptch1*^*−/−*^ MEFs the coimmunoprecipitation was realized with anti-Maml1 (D3E9) (cat.#11959; Cell Signaling, Danvers, MA, USA) or normal rabbit IgG (cat.#sc-2027; Santa Cruz Biotechnology) as control for overnight at 4 °C; the pre-clearing step and precipitation of complexes were realized with Protein A-Agarose (cat.#sc-2001; Santa Cruz Biotechnology). For immunoblot analysis the following primary antibodies were used: anti-Gli1 (L42B10) (cat.#2643), anti-Maml1 (D3E9) and anti-Notch1 (Val 1744) (D3B8) (cat.#4147) purchased from Cell Signaling; anti-Flag (cat.#F3165), anti-Myc (cat.#M4439), anti-Notch2 (Val1697) (cat.#SAB450200) and anti-β-Actin (cat.#A5441) from Sigma-Aldrich; anti-HA (cat.#sc-7392), anti-α-Tubulin (cat.#sc-8035) and anti-Lamin B (M20) (cat.#sc-6217) from Santa Cruz Biotechnology; anti-Gli2 (cat.#AF3635) from R&D Systems (Minneapolis, MN, USA). Bound antibodies were detected with enhanced chemiluminescence (ECL kit; Amersham, GE Healthcare, Lafayette, CO, USA). The intensity of protein expression was quantified using Quantity One Analysis Software (Bio-Rad). Values were normalized to housekeeping protein expression and represented as relative levels with respect to control sample.

### Chromatin immunoprecipitation

ChIP was performed as described earlier.^57, 58^ Immunoprecipitated DNA from HEK293T with Gli1 (H300) (cat.#sc-20687; Santa Cruz Biotechnology) or Maml1 (D3E9) or IgG (normal rabbit IgG) antibodies was eluted and analyzed by semiquantitative PCR, using a primer set encompassing two predicted binding sites for Gli zinc finger family (V$GLIF Matrix Family) (from −538 to −521; from −461 to −445, corresponding to dark circles in Figure 1f, low panel), on human *Patched1* promoter (GXP_227868, from −891 to −87 relative to start codon). Human *Patched1* promoter was identified using MatInspector (Software GmbH, Munich, Germany). The primer set used to specifically amplify Gli binding sites is the following: 5′-GAACCCAGCAGCCAGAGC-3′ and 5′-CGACCCCTTCACTGCAGAA-3′.

### Immunostaining and confocal imaging

Immunofluorescence staining of HEK293T cells was performed as described elsewhere.^51^ Twenty-four hours after transfection, the cells were stained with primary antibody: mouse anti-HA (cat.#MMS-101 P; Covance, Princeton, NJ, USA) and rabbit anti-Flag (cat.#F7425; Sigma-Aldrich). The secondary antibodies used were Alexa Fluor 594- and 488-conjugated respectively anti-mouse and anti-rabbit (Molecular Probes, Life Technologies). Nuclei were counterstained with Hoechst reagent. Single plane confocal images in the center of the cell were acquired using an inverted Olympus iX73 microscope equipped with an X-light Nipkow spinning-disk head (Crest Optics, Rome, Italy) and Lumencor Spectra X Led illumination. Images were collected using a CoolSNAP MYO CCD camera (Photometrics, Tucson, AZ, USA) and MetaMorph Software (Molecular Device, Sunnyvale, CA, USA) with a × 60 oil objective.

### Proximity ligation assay

*In situ* PLA was performed in NIH3T3 cells using the Duolink *In situ-Fluorescence* Technology, Olink Bioscience (Sigma-Aldrich). All the steps were performed according to the manufacturer’s protocol. Primary antibodies: anti-Gli1 (H300) and anti-Maml1 (N-20) (cat.#sc-18506) from Santa Cruz Biotechnology. Hybridization between the two PLA anti-rabbit PLUS and anti-goat MINUS probes leading the fluorescent red signal only occurs when the distance between the two antigens is less than 40 nm. In control experiment, cells were incubated with only one primary antibody and no significant binding was detected (only Gli1; only Maml1). Single plane confocal images were acquired using an inverted Olympus iX73 confocal microscope as described in immunofluorescence staining.

### Immunohistochemistry

Cerebella from E18.5 and E19.5 mice were collected and tissues were fixed in 4% formalin and paraffin embedded. Consecutive sections (2 *μ*m thick) were stained with H&E. Immunocytochemical assay was performed using an anti-PCNA antibody (cat.#ab15497; Abcam, Cambridge, UK). Detection was carried out with Mouse-to-Mouse HRP (DAB) staining system (cat.#MTM001-IFU; ScyTek Laboratories, Logan, UT, USA), according to the manufacturer’s instructions. Images were acquired with a Leica DM1000 microscope equipped with a ProgRes Speed XT^core^ 3 CCD camera and collected using ProgRes CapturePro 2.8 software (Jenoptik Optical Systems GmbH, Jena, Germany). Proliferation index was deduced by the count of PCNA-positive GCPs/total GCPs in EGL of wild type and *Maml1*^*−/−*^ cerebella. The midsagittal area and perimeter of cerebella were measured from pictures captured using Aperio ImageScope (Aperio, Leica Biosystems, Wetzlar, Germany) image analysis software.

### Cytofluorimetric analysis

Freshly isolated GCP cells from cerebellum were stained and analyzed on a FACS-Calibur with CellQuest software (BD Biosciences, San Jose, CA, USA). For Gli1 intracellular staining, BD Fixation/Permeabilization kit was used (cat.#554714; BD Biosciences) and cells were incubated with anti-Gli1 antibody (L42B10) (cat.#2643; Cell Signaling) or normal mouse IgG (cat.#sc-2025; Santa Cruz Biotechnology), used as a negative control.

### Statistical analysis

All results were reported as the mean±S.D. of at least three independent experiments, each performed in triplicate. Student’s *t-*test for unpaired samples was used to assess differences among groups. A *P*-value <0.05 was considered statistically significant (NS *P*>0.05; *P≤0.05; **P≤0.01; ***P≤0.001; ****P≤0.0001).
