# Supplementary material for: Maml1 acts cooperatively with Gli proteins to regulate sonic hedgehog signaling pathway
Source: Cell Death Dis. 2017 Jul 20;8(7):e2942–. doi: 10.1038/cddis.2017.326 (PMC5550871; doi:10.1038/cddis.2017.326)
Supplement: Supplementary Information [file cddis2017326x1.pdf]

Supplementary Figure S1 Quaranta R., et al.

a

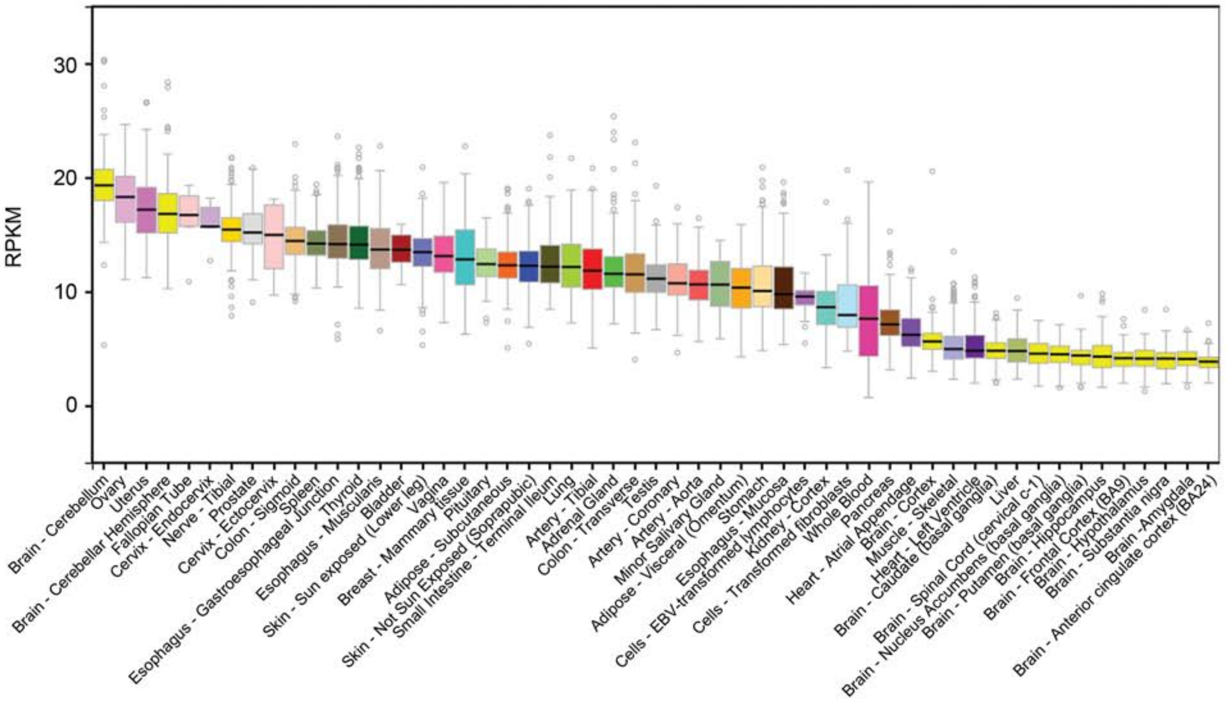

b

Gene	Design element	'pediatric high grade glioma' vs 'normal'	'adult high grade glioma' vs 'normal'	'atypical teratoid/ rhabdoid tumor' vs 'normal'	'group 3 medulloblastoma' vs 'normal'	'group 4 medulloblastoma' vs 'normal'	'pilocytic astrocytoma' vs 'normal'	'posterior fossa group A ependymoma' vs 'normal'	'posterior fossa group B ependymoma' vs 'normal'	'sonic hedgehog group medulloblastoma' vs 'normal'	'supratentorial ependymoma' vs 'normal'
maml1	202360_at				1					1.2	

***In silico* analysis of Maml1 expression in physiological and pathological conditions**

(a) RNA-seq of human tissue samples as indicated in Baseline Expression Atlas [http://www.ebi.ac.uk/gxa; experiment name: 53 GTEx; accession number: E-MTAB-2919; release 22.06.2016]. Cerebellum shows an high expression of Maml1 transcripts, as reported in the boxplot from Genotype-Tissue Expression (GTEx) Project website. RPKM=reads per kilobase per million mapped reads

(b) The table shows data for the expression of probe set 202360_at, representing the *Maml1* gene, from gene expression profile of pediatric and adult brain tumor types and normal brain, as reported in Differential Atlas database [array design: Affymetrix GeneChip Human Genome U133 Plus 2.0 (HG-U133_Plus_2); accession number: E-GEOD-66354]. A red box indicates that the gene was up-regulated in the test condition. The color intensity of filled boxes in the table represents how large the log_2 fold-change is for the gene respect to control: the larger the log_2 fold-change, the more intense the red color.

Supplementary Figure S2 Quaranta R., et al.

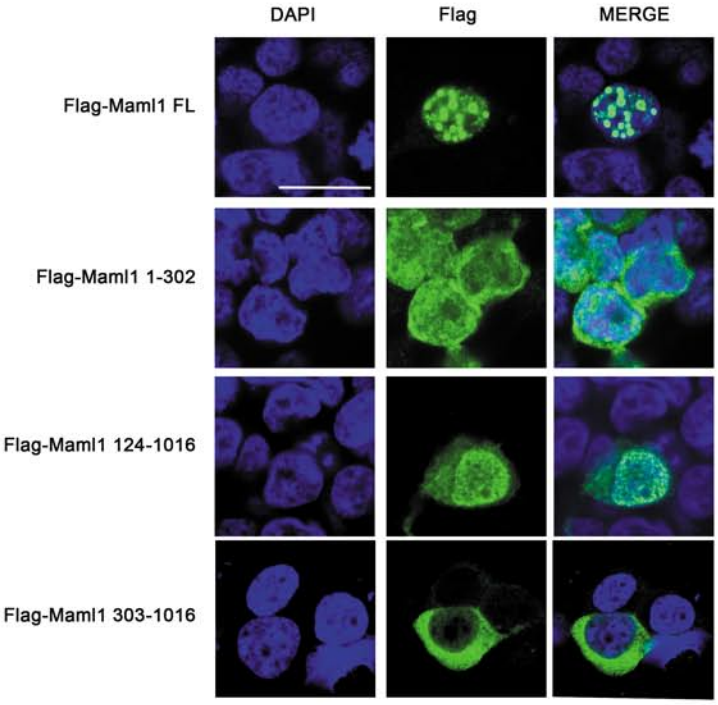

Subcellular distribution of transiently expressed Flag-tagged Maml1 FL and mutant forms

Representative single plane immunofluorescence images of HEK293T cells transfected with Flag-Maml1 full-length or mutant vectors, as indicated in Figure. Flag- (green) tags were visualized by confocal microscopy. Images were acquired as a single plane confocal images in the center of the cell that were captured using a 60x oil objective as described in M&M. Figure shows that Maml1 FL is able to move preferentially into the nucleus, in particular into the nuclear bodies. On the contrary, Maml1 mutant forms present a different subcellular localization: Maml1 1-302 is localized into the nucleus and also in the cytoplasm compartment; Maml1 124-1016 truncated form is present mostly into the nucleus, in a diffuse manner; Maml1 303-1016 (containing the TAD2, but without the NLS region) is preferentially located in the cytoplasm. Nuclei were DAPI-labeled (blue). Scale bar: 15 μm.

Supplementary Figure S3 Quaranta R., et al.

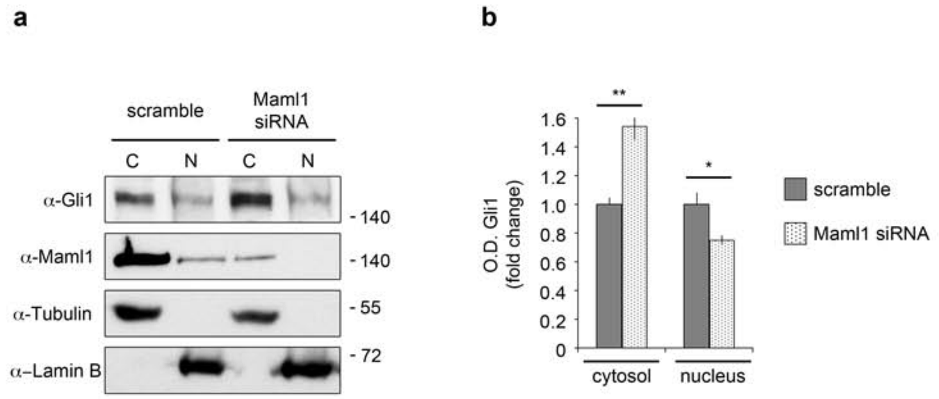

Maml1 influences the subcellular localization of Gli1

(a) Immunoblot analysis of cytosolic (C) and nuclear (N) protein fractions from wild-type MEFs Maml1-silenced, compared to scramble control with the indicated antibodies. Anti–Lamin B and anti-Tubulin were used as quality control of fractionated protein extracts and as loading control.

(b) Optical densitometry (O.D.) analysis of Gli1 protein expression evaluated by immunoblotting, related to panel a.

Supplementary Figure S4 Quaranta R., et al.

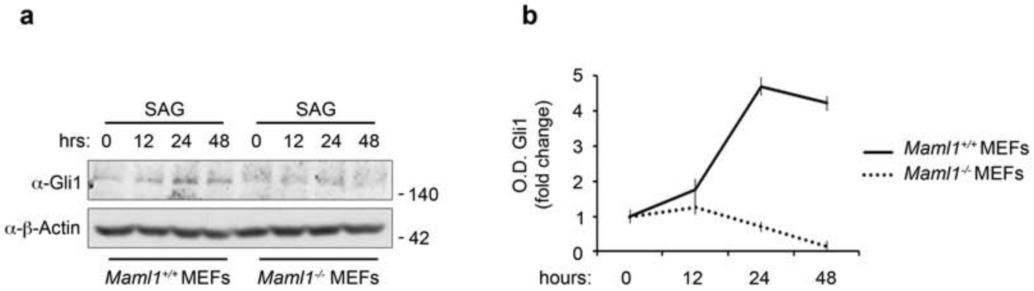

Maml1 depletion determines an impaired Shh signaling activation

(a) Gli1 expression in WCE prepared from *Maml1*$^{-/-}$ and control MEFs, treated with SAG for the indicated times, was detected by immunoblot.

(b) Optical densitometry (O.D.) analysis of Gli1 protein expression evaluated by immunoblotting, related to panel a.

Supplementary Figure S5 Quaranta R., et al.

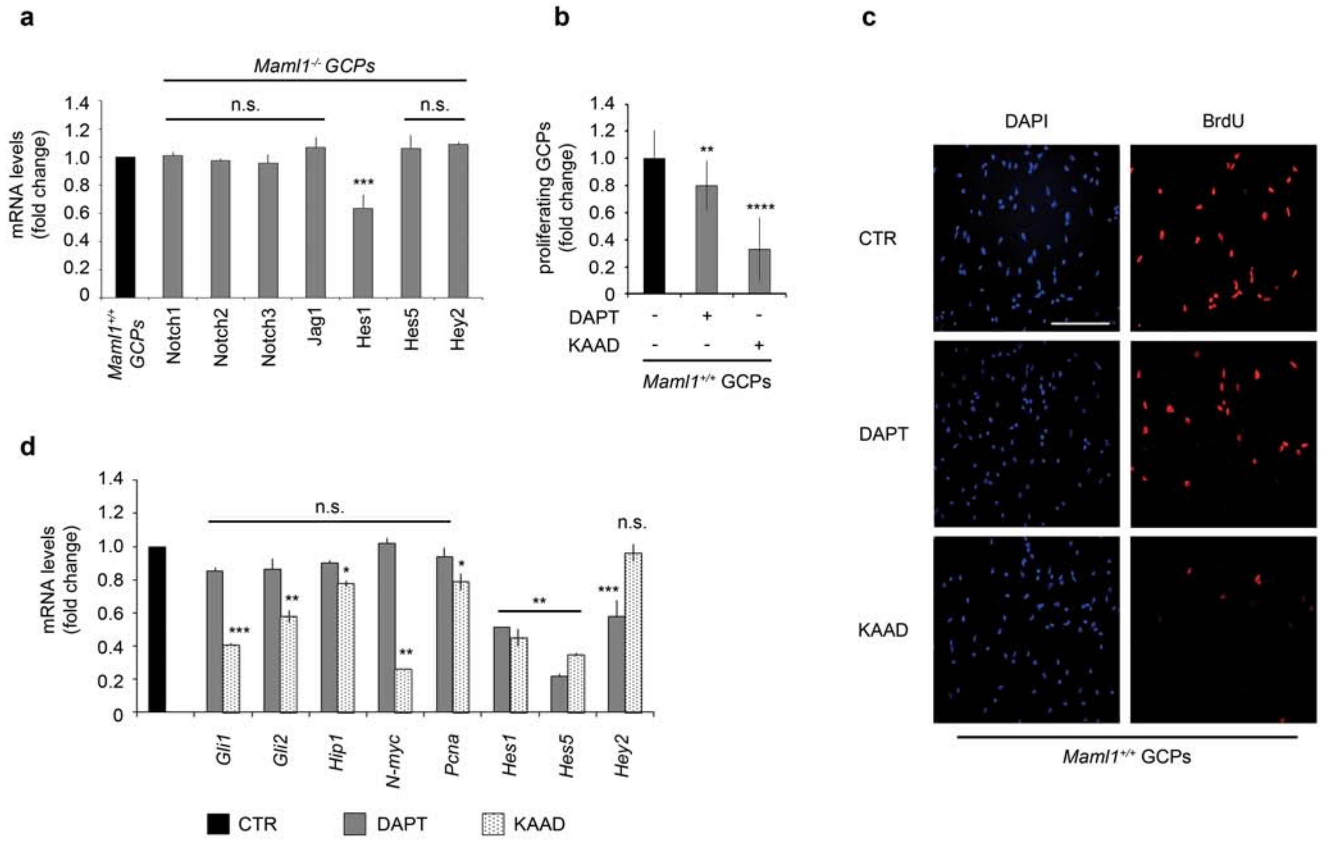

Compared effects on GCPs proliferation after Notch and Shh inhibition

(a) qRT–PCR analysis shows mRNA basal expression level of all components of Notch signaling pathway in GCP cultures from E19.5 *Maml1*$^{-/-}$ and control mice.

(b, c) BrdU incorporation assay in DAPT or KAAD-treated *Maml1*$^{+/+}$ GCPs at E19.5 after a 24h BrdU pulse. Mitotic index was calculated by number of BrdU-positive GCPs/total GCPs. The results were analyzed as fold of reduction respect to control cells (b). Proliferating cells are visualized in red; nucleus was labeled in blue. Scale bar: 200 μm (c).

(d) qRT–PCR analysis shows mRNA expression level of Notch and Shh target genes in GCP cultures from *Maml1*$^{+/+}$ mice after DAPT or KAAD treatment. The results were analyzed as fold change compared to control cells.

Data represent mean ± S.D. n.s. (not significant); * $p \leq 0.05$; ** $p \leq 0.01$; *** $p \leq 0.001$

Supplementary Figure S6 Quaranta R., et al.

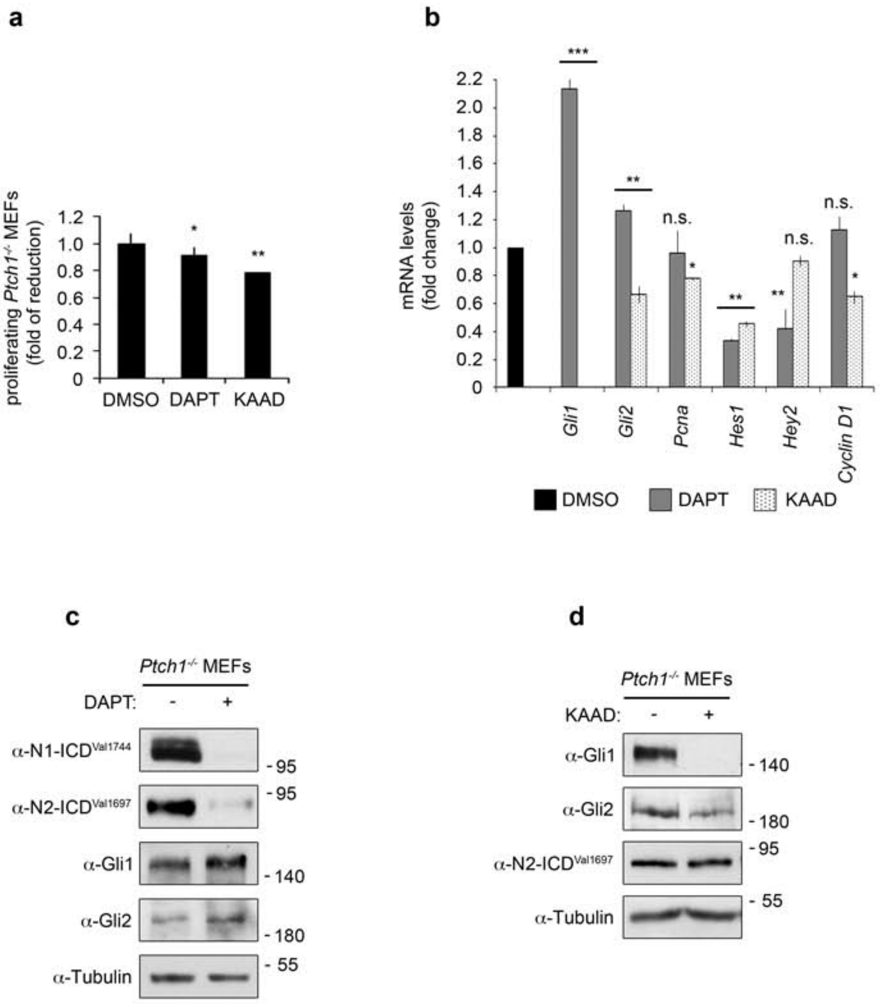

Compared effects on Ptch1$^{-/-}$ MEF proliferation after Notch and Shh inhibition

(a) MTT cell proliferation assay in *Ptch1$^{-/-}$* MEF cells after DAPT or KAAD treatment. The data are presented as fold of reduction respect to the control (DMSO).

(b) qRT–PCR analysis shows mRNA expression level of Shh and Notch target genes in *Ptch1$^{-/-}$* MEF cells after DAPT or KAAD treatment. The results were analyzed as fold change compared to control cells (DMSO).

(c, d) Immunoblot analysis of whole cell extracts from *Ptch1$^{-/-}$* MEFs using the indicated antibodies, after DAPT (c) or KAAD (d) treatment.

Data represent mean ± S.D. n.s. (not significant); * $p \leq 0.05$; ** $p \leq 0.01$; *** $p \leq 0.001$

Supplementary Table S1 Quaranta R. et al.

Species	Gene name	Symbol	RefSeq	Assay ID
Mouse	Cyclin D1	Ccnd1	NM_007631	Mm00432359_m1
Mouse	Cyclin D2	Ccnd2	NM_009829	Mm00438070_m1
Mouse	GLI-Kruppel family member GLI1	Gli1	NM_010296	Mm00494654_m1
Mouse	GLI-Kruppel family member GLI2	Gli2	NM_001081125	Mm01293117_m1
Mouse	Hairy and enhancer of split 1	Hes1	NM_008235	Mm01342805_m1
Mouse	Hairy and enhancer of split 5	Hes5	NM_010419	Mm00439311_g1
Mouse	Hairy/enhancer-of-split related with YRPW motif 2	Hey2	NM_013904	Mm00469280_m1
Mouse	Huntingtin interacting protein 1	Hip1	NM_146001	Mm00524503_m1
Mouse	Hypoxanthine guanine phosphoribosyl transferase	Hprt	NM_013556	Mm01545399_m1 (FAM)
Mouse	Hypoxanthine guanine phosphoribosyl transferase	Hprt	NM_013556	Mm00446968_m1 (VIC)
Mouse	Insulin-like growth factor 2	Igf2	NM_001122736	Mm00439564_m1
Mouse	Jagged1	Jagged1	NM_013822	Mm00496902_m1
Mouse	Mastermind-like 1	Maml1	NM_175334	Mm00614627_m1
Mouse	v-myc myelocytomatosis viral related oncogene, neuroblastoma derived	MycN	NM_008709	Mm00476449_m1
Mouse	Notch1	Notch1	NM_008714	Mm00435235_m1
Mouse	Notch2	Notch2	NM_010928	Mm00803077_m1
Mouse	Notch3	Notch3	NM_008716	Mm01345646_m1
Mouse	Paired box 6	Pax6	NM_001244198	Mm00443081_m1
Mouse	Proliferating cell nuclear antigen	Pcna	NM_011045	Mm00448100_g1
Mouse	Patched homolog 1	Ptch1	NM_008957	Mm00436026_m1

Quantitative real time PCR oligonucleotide sequences.
TaqMan Assay on Demand (Life Technologies, Applied Biosystems, Carlsbad, CA) code for the indicated genes.
